# BAFF Mediates Splenic B Cell Response and Antibody Production in Experimental Chagas Disease

**DOI:** 10.1371/journal.pntd.0000679

**Published:** 2010-05-04

**Authors:** Daniela A. Bermejo, María C. Amezcua-Vesely, Carolina L. Montes, María C. Merino, Ricardo C. Gehrau, Hugo Cejas, Eva V. Acosta-Rodríguez, Adriana Gruppi

**Affiliations:** Department of Immunology, School of Chemical Sciences, National University of Córdoba, Córdoba, Argentina; New York University School of Medicine, United States of America

## Abstract

**Background:**

B cells and antibodies are involved not only in controlling the spread of blood circulating *Trypanosoma cruzi*, but also in the autoreactive manifestations observed in Chagas disease. Acute infection results in polyclonal B cell activation associated with hypergammaglobulinemia, delayed specific humoral immunity and high levels of non-parasite specific antibodies. Since TNF superfamily B lymphocyte Stimulator (BAFF) mediates polyclonal B cell response *in vitro* triggered by *T. cruzi* antigens, and BAFF-Tg mice show similar signs to *T. cruzi* infected mice, we hypothesized that BAFF can mediate polyclonal B cell response in experimental Chagas disease.

**Methodology/Principal Findings:**

BAFF is produced early and persists throughout the infection. To analyze BAFF role in experimental Chagas disease, Balb/c infected mice were injected with BR3:Fc, a soluble receptor of BAFF, to block BAFF activity. By BAFF blockade we observed that this cytokine mediates the mature B cell response and the production of non-parasite specific IgM and IgG. BAFF also influences the development of antinuclear IgG and parasite-specific IgM response, not affecting *T. cruzi*-specific IgG and parasitemia. Interestingly, BAFF inhibition favors the parasitism in heart.

**Conclusions/Significance:**

Our results demonstrate, for the first time, an active role for BAFF in shaping the mature B cell repertoire in a parasite infection.

## Author Summary

Chagas disease, caused by the protozoan *Trypanosoma cruzi*, is endemic in Central and South America. It affects 20 million people and about 100 million people are at risk of infection in endemic areas. Some cases have been identified in non-endemic countries as a consequence of blood transfusion and organ transplantation. Chagas disease presents three stages of infection. The acute phase appears one to two weeks after infection and includes fever, swelling around the bite site, enlarged lymph glands and spleen, and fatigue. This stage is characterized by circulating parasites and many immunological disturbances including a massive B cell response. In general, the acute episode self-resolves in about 2 months and is followed by a clinically silent indeterminate phase characterized by absence of circulating parasites. In about one-third of the cases, the indeterminate phase evolves into a chronic phase with clinically defined cardiac or digestive disturbances. Current knowledge suggests that the persistence of parasites coupled with an unbalanced immune response sustain inflammatory response in the chronic stage. We believe that an effective treatment for chronic Chagas disease should combine antiparasitic drugs with immunomodulators aimed at reducing inflammation and autoreactive response. Our findings enlighten a new role of BAFF-BAFF-R signaling in parasite infection that partially controls polyclonal B cell response but not parasitespecific class-switched primary effectors B cells.

## Introduction

Chagas disease is a chronic disease caused by infection with *Trypanosoma cruzi*. Initially, the disease goes through an acute episode which is characterized by circulating parasites and immunological disturbances that, at the level of B cell compartment, are mature and immature B cell apoptosis [1], [2] as well as massive B cell response [3]. This polyclonal response [4]–[6], which results in hipergammaglobulinemia [7] and delayed parasite specific humoral response [8], [9], may be the original cause for the autoimmune phenomena that have been described in chronic phases of infection [10], [11]. It is well known that polyclonal activation of B lymphocytes is invariably accompanied by autoantibody production [12], [13].

The polyclonal B cell activation that occurs during infection, induced by the host response (T cell response, cytokines) or parasite antigens, may disrupt normal immune regulatory mechanisms and cause autoimmunity [14] It has been reported that polyclonal B cell response in *T. cruzi* infected mice is predominantly helper T-cell dependent [15]. However, Ig-secreting plaque-forming cells are recorded in athymic (nude) mice after *T. cruzi* infection [5] suggesting that T-independent mechanisms can also mediate polyclonal B cell response. Several parasite-encoded proteins have been identified as B cell mitogens [13], [16]–[18] and some of these *T. cruzi* antigens trigger *in vitro* polyclonal B cell activation and differentiation in a T-independent way [16], [17]. We have reported that macrophages from normal mice cultured with *T. cruzi* glutamate dehydrogenase, a T-independent type II polyclonal B cell activator, secrete high level of BAFF that mediates B cell polyclonal activation [17], suggesting that BAFF may mediate the polyclonal B cell response *in vivo* during *T. cruzi* infection.

BAFF is a crucial factor for the survival of peripheral B cells [19]–[21]. But, in excess, BAFF leads to the development of autoimmune disorders in animal models. It has been described that BAFF transgenic mice show clear signs of B cell hyperplasia and hyperglobulinemia. These mice have enlarged spleen, Peyer's patches and lymph nodes, circulating immune complexes, rheumatoid factors, and anti-DNA Abs [22]. In addition, high levels of BAFF have been detected in the serum of patients with various autoimmune disorders [23], [24]. Based on the fact that BAFF transgenic and *T. cruzi* infected mice share many immunological features like polyclonal activation, autoantibody production and autoimmunity, we hypothesized that BAFF can participate in the polyclonal B cell response observed in experimental Chagas disease. In the present study, we quantified the levels of BAFF and analyzed the participation of BAFF on B cell response by blocking its activity with a soluble BAFF-receptor in *T. cruzi* infected mice.

## Methods

### Infection with *T. cruzi* and treatment with BR3:Fc or control IgG2a

BALB/c mice were originally obtained from School of Veterinary, La Plata National University (La Plata, Argentina) and housed in our animal facility where all experiments were performed in compliance with the Institutional Review Board and Ethical Committee of the School of Chemical Sciences, National University of Cordoba. BALB/c mice 6–8 wk old were intraperitoneally (i.p.) infected with 500 trypomastigotes from *T. cruzi* (Tulahuén strain) diluted in physiological solution, as previously described [2], [25]. Non-infected normal littermates were injected i.p. with physiological solution and processed in parallel. For BAFF activity blocking, one day after infection, mice were injected i.p. with 150 ug of BR3:Fc (Genentech Inc., South San Francisco, CA, USA) three times per week. As control, infected mice were injected with 150 ug of IgG2a or physiological solution. Non-infected normal littermates were injected i.p. with physiological solution and injected i.p. with 150 ug of BR3:Fc or 150 ug of IgG2a or physiological solution with the same schedule described above and processed in parallel. At 15 days after infection, mice (number indicated in each figure) were killed by cervical dislocation, blood was collected and lymphoid organs were removed.

BR3:Fc efficacy of BAFF neutralization was tested *in vivo* evaluating the reduction of splenic B cell subsets according to Lin *et al* [26]. Also, BR3:Fc neutralizing BAFF activity was evaluated in an *in vitro* assay measuring IgA concentration in the supernatant of peritoneal B cells cultured with CpG plus recombinant BAFF [27], [28] in presence or in absence of BR3:Fc (data not shown).

### Parasitemia counts

Blood was collected by retro-orbital bleeding, erythrocytes were lysed in a 0.87% ammonium chloride buffer, and viable trypomastigotes counted in a Neubauer counting chamber [2].

### Cell preparation

Spleen and inguinal lymph nodes were obtained and homogenized through a tissue strainer. Peritoneal cells were obtained by peritoneal washouts and bone marrow cells were isolated by flushing femurs and tibias of mice with RPMI 1640. When it was necessary, red blood cells were lysed for 5 min in Tris-ammonium chloride buffer. Viable mononuclear cell numbers were determined by trypan blue exclusion using a Neubauer counting chamber. Cell suspensions were processed for Flow cytometry studies or culture as indicated below.

### Purification of splenic cell population by cell sorting

To obtain B cells, T cells, dendritic cells and F 4/80^+^ macrophages, splenic cells from infected mice were stained with anti-B220 APC, anti-CD3 FITC, anti-CD11c PE, anti-F4/80 Biotin followed by Streptavidin Per-CP purchased from BD, and sorted by positive selection with FACSAria Cell Sorter (Becton Dickinson) to enrich populations to 98% for B and T cells and 88% for CD11c^+^ and F 4/80^+^.

### Reverse transcription of mRNA and its relative quantification by real time

Cells were incubated with TRIzol reagents (Life Technologies) and RNA was extracted according to the manufacturer's recommendation and stored at −70°C. RNA was reverse transcribed using Moloney murine leukemia virus reverse transcriptase (Invitrogen, USA) at 42°C for 60 min. One microgram of RNA was used to generate first cDNA strain. Real Time PCR reactions for mouse BAFF and β-Actin detection were performed using the following primers pairs: BAFF (Mm00446347_m1, Applied Biosystems) and HPRT (HPRT-F: 5′-AAGCTTGCTGGTGAAAAGGA-3′; and HPRT R: 5′-TCCAACAAAGTCTGGCCTGT-3′). The reaction mixtures contained: TaqMan Universal PCR Master Mix in the case of BAFF or 2X SYBR Green PCR Master Mix, 800 nM of HPRT primers, and 50 ng of cDNA. All reactions were performed in triplicate and were cycled as follows: 95°C for 10 min, 1 cycle; 95°C 15 s, 60°C 1 min, 40 cycles; 95°C 15 s, 1 cycle, 60°C 1 min, 1 cycle, followed by a melting curve rising from 60 to 95°C incrementally using in a 7500 System apparatus (Applied Biosystems). HPRT was used as standard to normalize cDNA loading [29].

### Flow cytometry studies

Cell suspensions were washed twice in ice-cold FACS buffer (Physiological solution with 2% fetal bovine serum (FBS, Gibco)) and preincubated with anti-mouse CD32/CD16 mAb (Fc block) for 30 min at 4°C. The cells were then incubated with each PE-, FITC-, or biotinylated Ab (e-Bioscience, San Diego, USA) for 30 min at 4°C and washed with FACS buffer: PE-anti B220, FICT-anti IgM and biotin-anti IgD to identify mature B cells; PE-anti CD138 and FICT-anti B220 to identify plasma cells; PE-anti B220, FICT-anti CD24 and biotin anti IgD to identify mature B cells in bone marrow, and biotin anti B220 to identify peritoneal B cells. Data were acquired on a FACSCanto II cytometer (Becton Dickinson) and analyzed using Flow Jo (Tree Star) software.

### Soluble BAFF determination

BAFF concentration (ng/ml) was determined in sera and culture supernatant of mononuclear cells from lymphoid organs from normal or *T. cruzi* infected mice by ELISA following manufacturer's instructions (Axxora, USA).

### Total immunoglobulin determination

Mononuclear cells from lymphoid organs from normal or *T. cruzi* infected mice treated with physiological solution or BR3:Fc or IgG2a control obtained at day 15 post infection (p.i.) were cultured with media for 30 h. IgM and IgG concentrations (ng/ml) were determined by ELISA as previously described [17]. In brief, plates were coated with 2.5 ug/ml of the type-specific goat anti-mouse Ab (IgM, IgG; Sigma-Aldrich Chemical Co) overnight at 4°C, and blocked with 1% Bovine serum albumin (BSA). Culture supernatants were incubated overnight at 4°C. Peroxidase-conjugated anti-mouse IgG or anti-mouse IgM (2.5 µg/ml) were added and incubated for 1 h at 37°C. The reaction was developed with TMB Substrate Reagent (BD OptEIA™). The concentration was measured with reference to standard curves using known amounts of the respective murine Ig (Sigma-Aldrich Chemical Co.).

### Parasite specific serum Ab determination

The titers of *T. cruzi* specific seric IgM and IgG were determined by ELISA [30], [31] using *T. cruzi* trypomastigotes recombinant Ags following manufacturer instruction (Wiener lab, Argentina). ELISA test sera were considered positive if the mean OD value was two standard deviations above the mean value for control sera assayed in parallel.

### ANA serum IgG determination

Amount of antinuclear specific IgG Ab (ng/ml) and IgG1, IgG2a and IgG3 (OD) was determined by ELISA using a ANA ELISA kit (Alpha Diagnostic, San Antonio, TX, USA) following the manufacturer's instructions in sera from *T. cruzi* infected mice treated with physiological solution or BR3:Fc or IgG2a control.

### Histopathology studies

Hearts were fixed in formaldehyde and embedded in paraffin blocks, after which 5 to 20-µm-thick transverse sections were mounted on slides and subsequently stained with hematoxylin and eosin. Photographs were taken using a Nikon Eclipse TE 2000 U equipped with a digital video camera. The grade of heart parasitism was evaluated analyzing the number of nests containing amastigotes per section.

### Statistical analysis

Statistical significance of comparisons of mean values was assessed by a two-tailed Student's *t* test or nonparametric Mann-Whitney *U* test using Graph pad software. In the case of *T. cruzi* specific Abs, mean values was assessed by a one way non parametric Kruskal-Wallis test. p≤0.05 was considered significant.

## Results

### BAFF concentration increased as a consequence of *T. cruzi* infection

**Figure 1:**
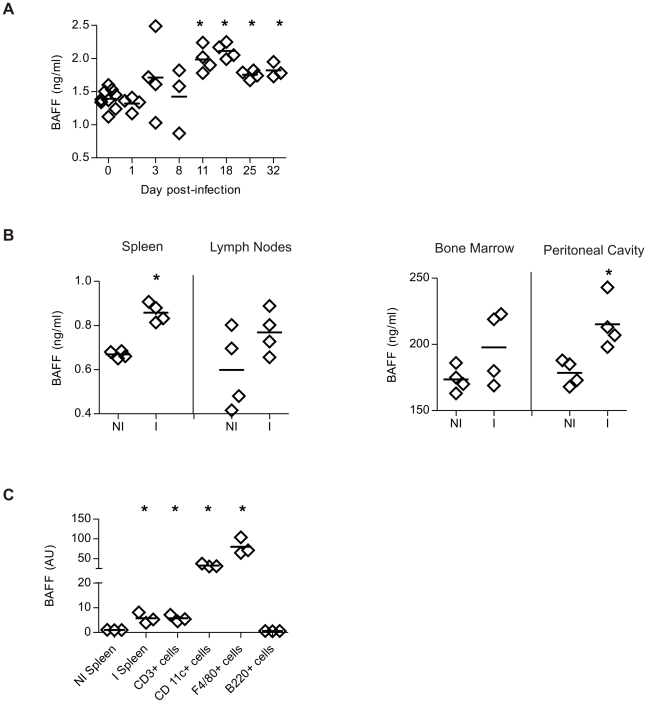
BAFF production in *T. cruzi* infected mice. BAFF concentration (ng/ml) was determined by ELISA in: **A**, Sera from non-infected (day 0) or *T. cruzi* infected mice obtained at different times of infection, **B**, culture supernatant of cells from spleen, bone marrow, lymph nodes and peritoneal cavity from non-infected (NI) or *T. cruzi* infected (I) mice obtained at 15 days p.i. **C**, Splenic CD3^+^, CD11c^+^, F4/80^+^ and B220^+^ cells from *T. cruzi* infected mice were purified by cell sorting. mRNAs were obtained from these populations from total splenic cells from I mice. Spleen cells from NI and I mice were used as control. BAFF mRNA levels were quantified by Real Time PCR. The amounts of transcripts were normalized to HPRT transcripts and were expressed relative to the amount in splenic cells from NI mice. Diamonds represent the value obtained from each mouse. The lines represent the media value in each analyzed group. *, p≤0.05. Results are representative for two individual experiments.

To investigate systemic BAFF concentration during *T. cruzi* infection, serum samples were collected at different times p.i. and analyzed for levels of circulating BAFF by ELISA. During an ongoing *T. cruzi* infection, a significant increase in the seric levels of BAFF was detected at 11 days p.i. BAFF concentration presented the highest value at the peak of parasitemia (day 11–15 p.i, [2]) and persisted elevated until the last day analyzed (day 32 p.i) (Fig 1A). Additionally, cells from the spleen and bone marrow, but not from the lymph nodes and peritoneal cavity, obtained from 15-day infected mice and cultured in the absence of stimuli, released higher concentrations of BAFF than cells from non-infected mice (Fig 1B).

To identify which mononuclear cells produced BAFF in *T. cruzi* infected mice, CD3^+^cells, CD11c^+^cells, F4/80^+^ cells and B220^+^ cells were obtained by cell sorting from the spleen of infected mice. By real-time PCR we determined that F4/80^+^ cells and CD11c^+^ cells showed high levels of mRNA coding for BAFF. The transcript for BAFF was present at low levels in T cells and undetectable in B cells. mRNA from total spleen cells from *T. cruzi* infected mice was used as positive control (Fig 1C).

### BAFF blockade resulted in a diminution of mature B cells in lymphoid organs

In order to analyze the potential role of BAFF in the massive B cell response in experimental Chagas disease, BAFF activity was blocked by injecting BR3:Fc, a soluble BAFF receptor, into *T. cruzi* infected mice. Considering that the different B cell compartments are dissimilarly affected by *T. cruzi* infection [2], [32], [33], we evaluated how the BAFF blockade affected each B cell compartment individually.

**Figure 2:**
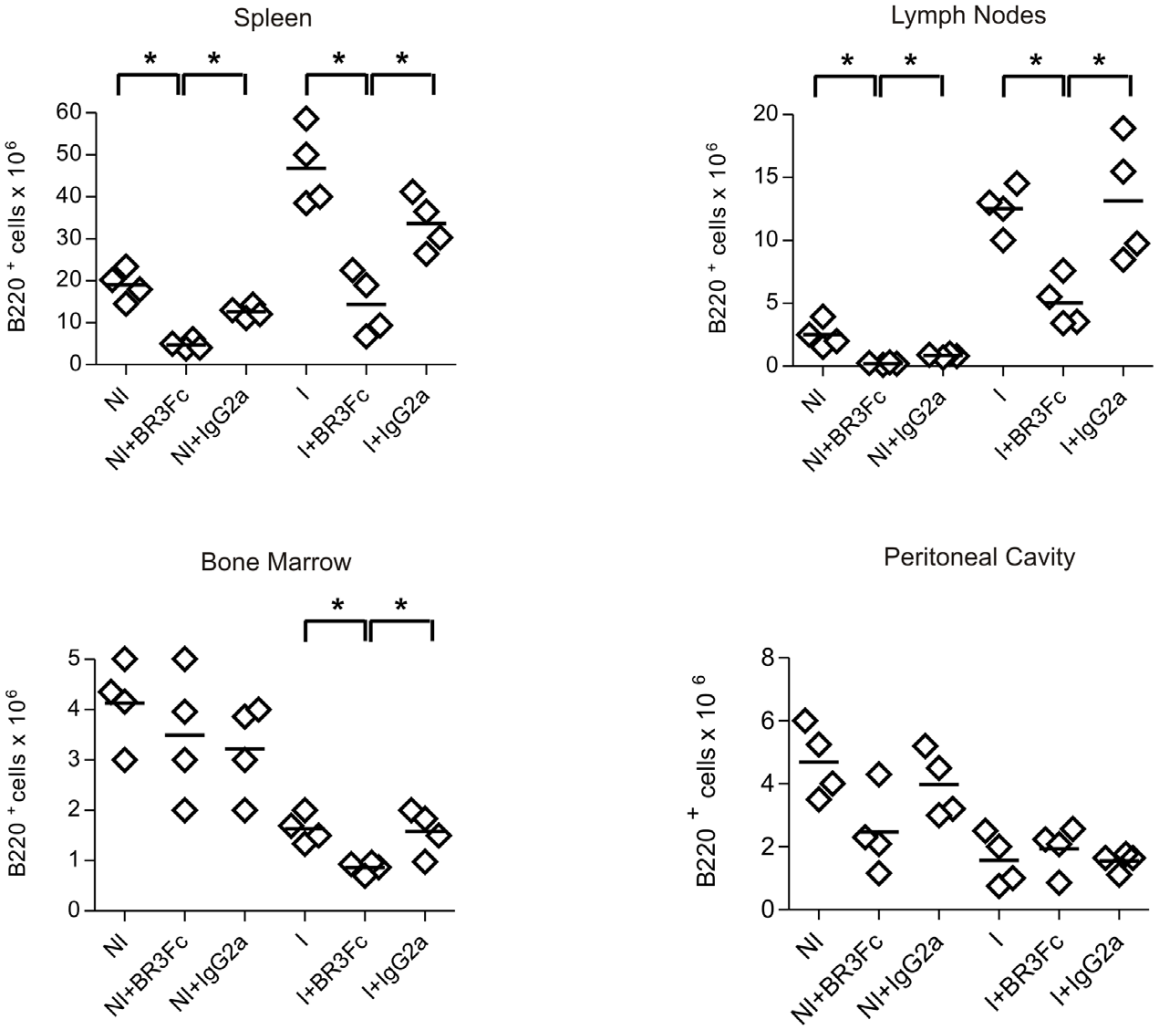
B cell number in non-infected or *T. cruzi* infected mice treated with BR3:Fc. Cells from spleen, lymph nodes, bone marrow and peritoneal cavity from non-infected (NI) or *T. cruzi* infected (day 15 p.i.) mice treated with physiological solution (I) or BR3:Fc (I+BR3:Fc) or IgG2a control (I+IgG2a) were obtained, stained with anti-B220 and analyzed by flow cytometry. Graphs show the number of B220^+^ cells in different lymphoid compartments. Diamonds represent the value obtained from each mouse. The lines represent the media value.*, p≤0.05. Results are representative for three individual experiments.

We observed that treatment with BR3:Fc resulted in a significant reduction of splenic and lymph nodes B220^+^ cell number in non-infected and in *T. cruzi* infected mice (Fig 2). We determined that the reduction affected mature B220^+^IgD^+^IgM^+^ spleen cells but did not induce significant changes in the number of B220^+^IgD^−^IgM^+^ spleen cells (phenotype compatible with immature, marginal zone and activated B cell) (Fig S1 A). BAFF inhibition reduced the already low number of B cells present in the bone marrow of *T. cruzi* infected but did not affect the number of B cells in the bone marrow of normal mice (Fig 2). This result could be explained by the fact that BAFF blockade affected mature B220^+^ IgD^+^CD24^+^ but not immature B220^+^ IgD^−^ CD24^hi^ B cells from bone marrow (Fig S1 B). Immature B cells are the major B cell population in the bone marrow of normal mice but are almost absent in *T. cruzi* infected mice that present only mature B cells [2]. In addition, we determined that BAFF blockade did not change the number of peritoneal B220^+^ cells observed in *T. cruzi* infected mice (Fig 2) that were reduced as a consequence of infection [34]. Mice injected with control isotype non-blocking Ab presented no significant difference in B cell number in comparison to non-treated non infected (NI) or infected (I) mice (Fig 2).

**Figure 3:**
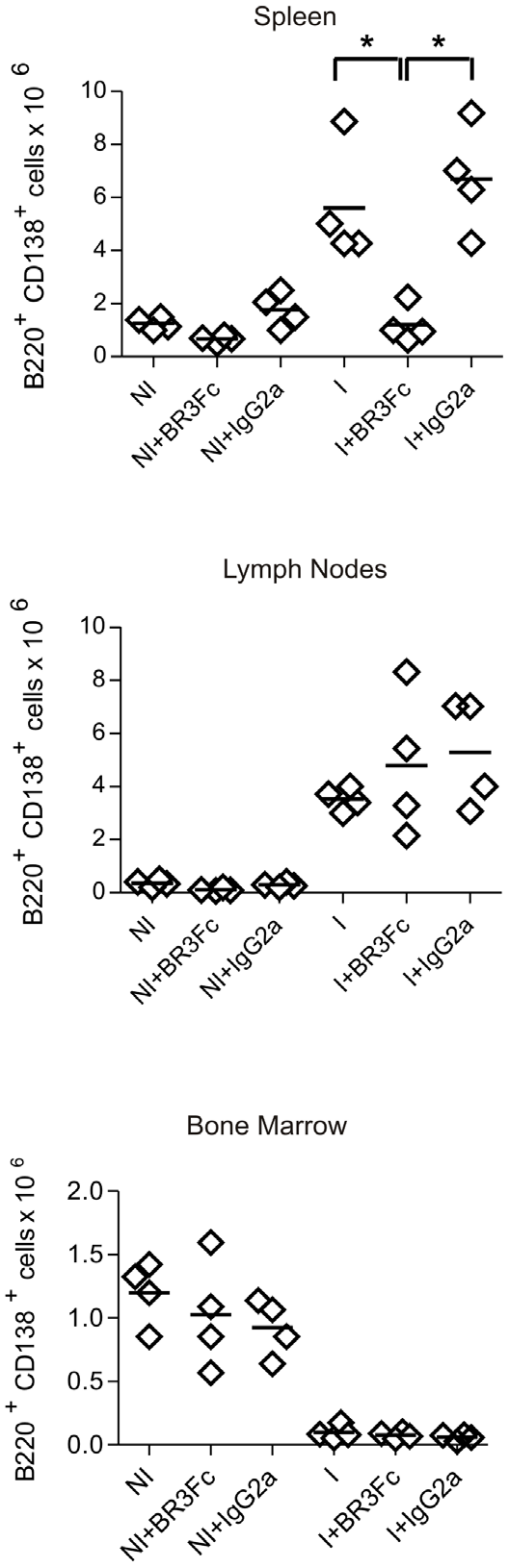
Plasma cell number in non-infected or *T. cruzi* infected mice treated with BR3:Fc. Cells from spleen, lymph nodes and bone marrow from non-infected (NI) or *T. cruzi* infected (day 15 p.i.) mice treated with physiological solution (I) or BR3:Fc (I+BR3:Fc) or IgG2a control (I+IgG2a) were obtained, stained with anti-B220 and anti-CD138 and analyzed by flow cytometry. Graphs show the number of B220^+^CD138^+^cells analyzed in each experimental group. Diamonds represent the value obtained from each mouse. The lines represent the media value in each condition.*, p≤0.05. Results are representative for three individual experiments.

Next, we analyzed if B cell reduction observed in lymphoid organs as a consequence of BAFF blockade also affected plasma cell number. B220^+^CD138^+^ cell number declined markedly in the spleen but remained unchanged in lymph nodes of BR3:Fc treated-*T. cruzi* infected mice (Fig 3). *T. cruzi* infection *per se* induced a severe reduction of plasma cell number in bone marrow (Fig 3) and no further reduction was observed after BAFF blockade.

### BAFF inhibition partly impairs humoral immune response in *T. cruzi* infected mice

**Figure 4:**
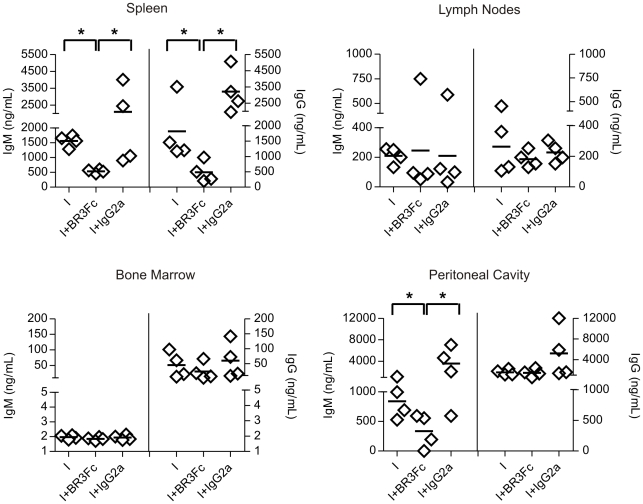
Immunoglobulin concentration in *T. cruzi* infected mice treated with BR3:Fc. Cells from spleen, lymph nodes, bone marrow and peritoneal cavity from *T. cruzi* infected (day 15 p.i.) mice treated with physiological solution (I) or BR3:Fc (I+BR3:Fc) or IgG2a control (I+IgG2a) were obtained and cultured with media without any stimulus for 30 h. IgM and IgG concentration (ng/ml) was determined in culture supernatants by ELISA. Diamonds represent the value obtained from each mouse. The lines represent the media value in each condition.*, p≤0.05. Results are representative for three individual experiments.

The reduction of plasma cell number in *T. cruzi* infected mice treated with BR3:Fc correlated with a significant reduction in IgM and IgG concentration in culture supernatant of splenic cells (Fig 4). Interestingly, while the concentration of IgG was maintained, a reduction of IgM concentration was detected in the culture supernatant of cells from peritoneum of *T. cruzi* infected mice with blocked BAFF activity. According to plasma cell number results, no differences were detected in IgM and IgG concentrations produced by cells from lymph nodes and bone marrow from treated and untreated infected mice (Fig 4).

**Figure 5:**
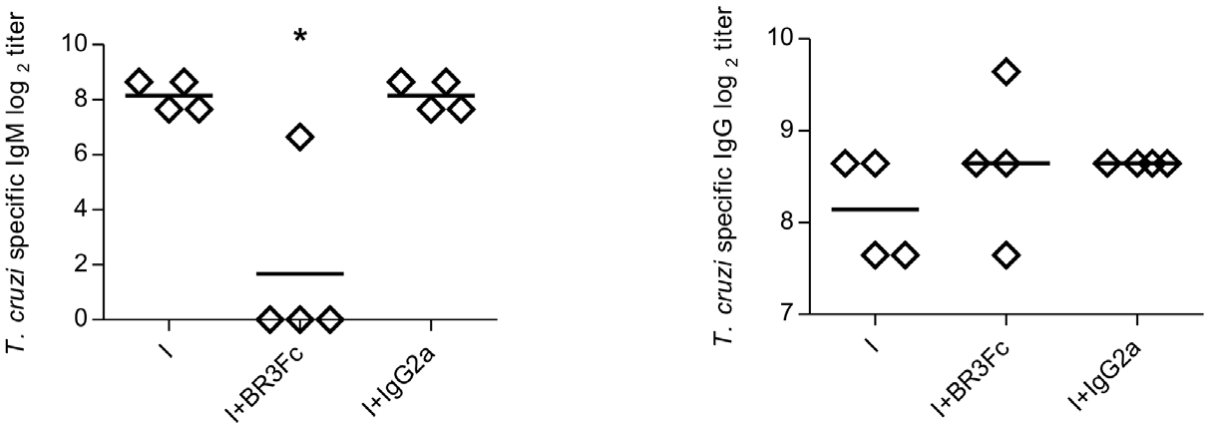
Parasite specific immunoglobulins in sera from *T. cruzi* infected mice treated with BR3:Fc. Sera from *T. cruzi* infected mice treated with physiological solution (I) or BR3:Fc (I+BR3:Fc) or IgG2a control (I+IgG2a) were obtained at day 15 p.i. and analyzed by ELISA to determine *T. cruzi* specific IgM and IgG Ab titers. Diamonds represent the value obtained from each mouse. The lines represent the media value in each case. *, p≤0.05. Results are representative for two individual experiments.

To analyze the biological significance of the reduced Ab response observed in BR3:Fc treated *T. cruzi* infected mice, we evaluated the levels of parasite specific Abs in sera. We observed that preventing BAFF binding drastically diminished trypomastigotes *T. cruzi-*specific IgM titers while *T. cruzi-*specific IgG titers did not change (Fig 5). Parasite-specific Abs were practically undetectable in the culture supernatants of the lymphoid organs obtained from infected mice indicating a low frequency of *T. cruzi* antigen-specific B cells as reported [8], [9] and a high frequency of non-parasite specific Abs (data not shown).

**Figure 6:**
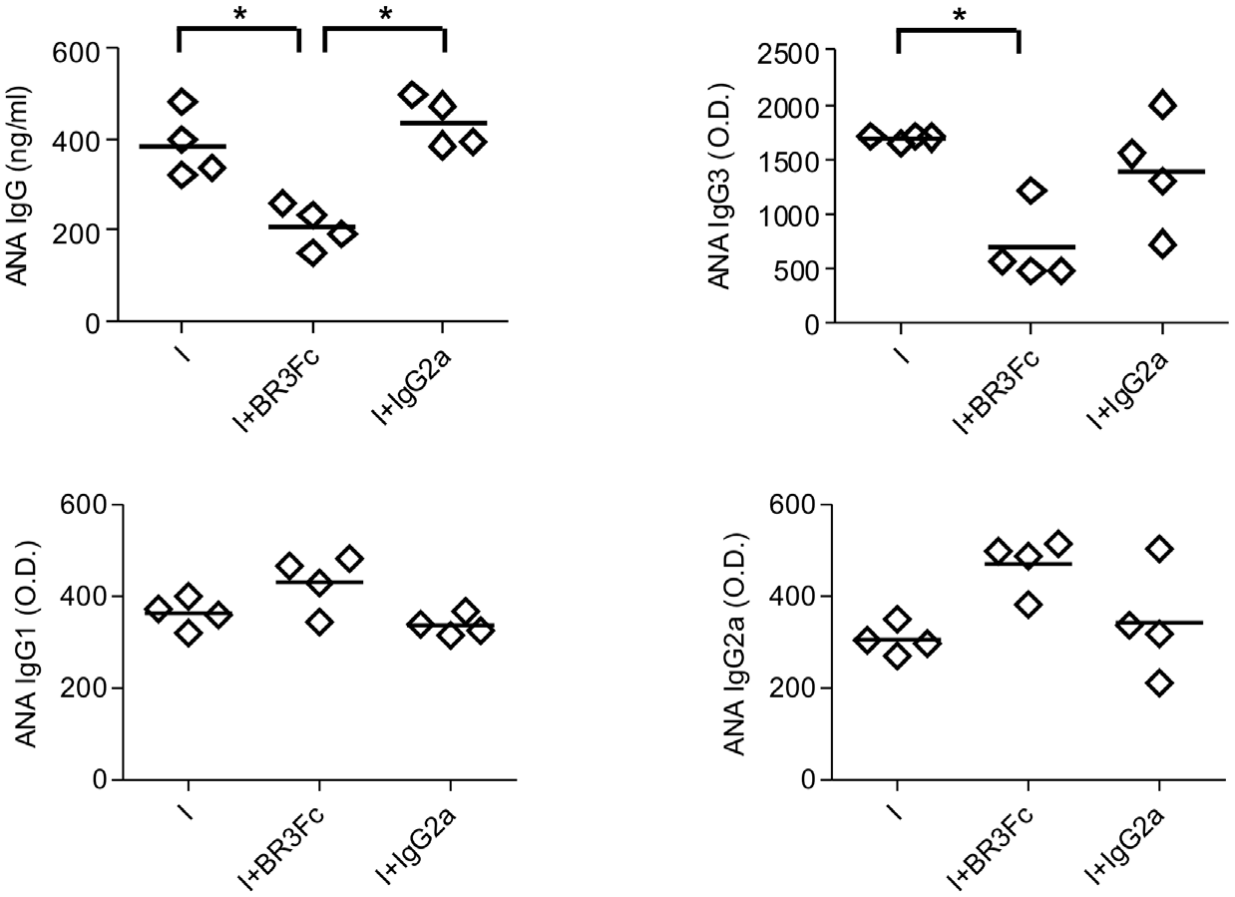
Autoreactive immunoglobulins in sera from *T. cruzi* infected mice treated with BR3:Fc. Sera from *T. cruzi* infected mice treated with physiological solution (I) or BR3:Fc (I+BR3:Fc) or IgG2a control (I+IgG2a) were obtained at day 15 p.i. and analyzed by ELISA to determine the levels of antinuclear (ANA) specific IgG (ng/mL) or ANA IgG3, IgG1 and IgG2a (O. D.) Diamonds represent the value obtained from each mouse. The lines represent the media value in each case. *, p≤0.05. Results are representative for two individual experiments.

To address the role of BAFF in the autoreactive humoral response, we evaluated autoreactive Abs in the sera of treated and control infected mice at 15 days p.i.. It has been described that autoreactive Abs such as anti-actin, anti-myosin, anti-myoglobin and antinuclear Abs (ANA) among others are present in the acute and chronic phase of the pathology [35]–[37]. However, we were unable to detect autoreactive Abs others than ANA at the acute phase of the infection (day 15 p.i.). Therefore, ANA were tested as markers of the effect of BR3:Fc treatment on the autoreactive B cell population during acute phase of infection. We observed that BAFF blockade prevented the production of ANA of IgG isotype in *T. cruzi* infected mice. The isotype of IgG involved in this reaction was IgG3 but not IgG2a or IgG1 (Fig 6).

### BAFF inhibition influences parasite replication in the heart

**Figure 7:**
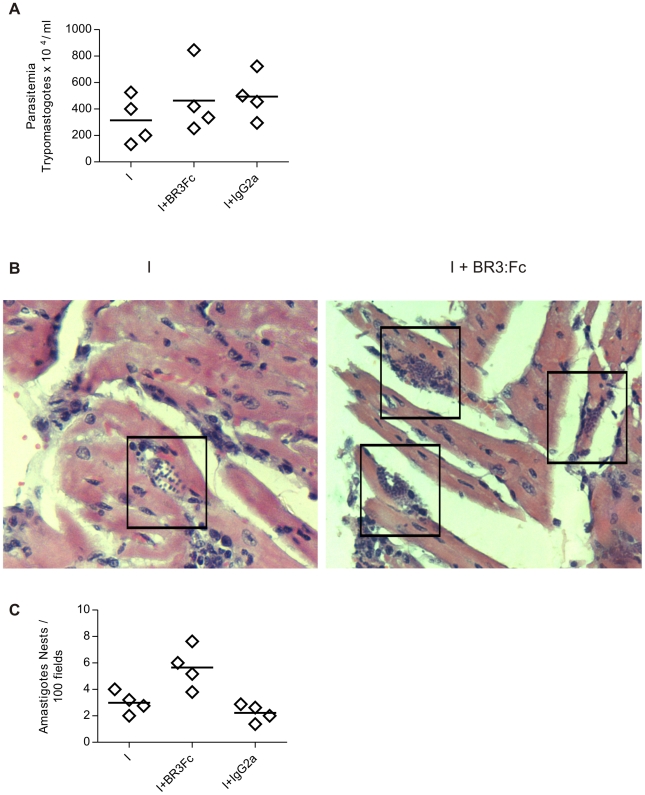
Circulating and tissue parasites in *T. cruzi* infected mice treated with BR3:Fc. **A**, Number of circulating parasites in blood samples from *T. cruzi* infected mice treated with physiological solution (I) or BR3:Fc (I+BR3:Fc) or IgG2a control (I+IgG2a) was determined at days 15 p.i. by counting in Neubauer chamber. **B**, Photomicrographs from heart sections obtained from *T. cruzi* infected mice treated with physiological solution (I) or BR3:Fc (I+BR3:Fc) stained with hematoxilina/eosina (400X). Amastigote nests are demarcated with squares. Inset shows one of them (1000X). **C**, Number of amastigote nests counted in 100 histological fields of hearts. Diamonds represent the value obtained from each mouse. The lines represent the media value in each day p.i. Results are representative for two individual experiments.

To analyze if B cell reduction and the decrease of IgM parasite-specific Abs affected the parasite replication, we measured the number of circulating trypomastigotes in blood of infected mice and the grade of tissue parasitism by evaluating amastigote niches in heart. Parasitemia was similar in *T. cruzi* infected mice treated with physiological solution, BR3:Fc or IgG2a control (Fig 7A), while cardiac parasitism was increased in BR3:Fc treated infected mice in comparison to untreated infected mice (Fig 7B,C). Thus, the hearts of infected mice in which BAFF activity was blocked had higher number of nest of amastigotes in the myocardial fibers of the auricle than non-treated infected mice (Fig 7B,C).

## Discussion

Most of the information about BAFF in disease is linked to autoimmune pathologies [38], but little information is available about BAFF in infectious diseases [39], [40]. Previously, we reported that BAFF is involved in the polyclonal B cell activation triggered *in vitro* by a *T. cruzi* antigen [17]. Here, we extended these data showing that BAFF increased early in *T. cruzi* infected mice and persisted at high levels throughout the infection. Our data show that cells from immune system as macrophages and dendritic cells which are susceptible to be infected by *T. cruzi* and in which the parasite can replicate intracellularly [41], [42] are an important source of BAFF. It has been reported that BAFF is mainly produced by innate immune cells such as neutrophils, macrophages, monocytes, dendritic cells (DCs) and follicular DCs [43] stimulated by cytokines often produced during inflammation and infections [43], [44], as well as by the Toll-like receptors ligands [45]. Then, it is possible that IL-10 and/or IFNγ, which increase during infection [46]–[48], as well as TLR ligands expressed by *T. cruzi* [49], [50] or parasite antigens [17] trigger BAFF secretion.

To analyze the role of BAFF in *T. cruzi* infection, infected mice were treated with a soluble BAFF-R, BR3:Fc, to block BAFF activity. The use of BAFF-R Fc considerably reduced mature peripheral B cell numbers in *T. cruzi* infected mice. As it was previously reported for normal mice [21], [51], [52], we observed that mature B cells from *T. cruzi* infected mice have different BAFF requirements than peritoneal mature B cells and/or immature B cells for their survival. Reduction in mature B cells was also observed in the bone marrow of infected mice, probably as a consequence of the diminution of peripheral mature B cells.

Following B cell depletion, BAFF blockade resulted in a reduction of splenic B220^+^CD138^+^ plasma cells. When we analyzed the proportion of plasma cells with respect to the number of total B cells we observed similar values in BR3:Fc treated or untreated infected mice (data not shown). These results suggest that during *T. cruzi* infection, BAFF apparently controls plasma cell numbers by reducing the pool of mature B cells. Concomitantly with plasma cell diminution, a reduction of total IgM and IgG production by splenic cells was observed in BR3:Fc treated infected mice. Since most of the Abs produced by splenic B cells are non-parasite specific [8], our results suggest that BAFF is regulating polyclonally activated B cells rather than antigen-specific activated B cells. In agreement, parasite-specific IgG titers were not affected by BAFF blockade while parasite-specific IgM almost disappeared in BR3:Fc treated infected mice. Similar behaviour was observed in three different independent experiments analyzed at 15 and 22 days p.i. Our results confirm and complete previously reported findings on the role of BAFF-BAFF-R signalling in the survival and maintenance of the mature B cell compartments [reviewed in 53], and that BAFF inhibition had a markedly small effect on IgG^+^ B cells and long-lived plasma cells. Scholz *et al* [54] reported that IgM-bearing memory cells are sensitive to BAFF depletion whereas IgG-bearing memory cells are not.

Chagas disease pathology is associated to autoimmunity [55]–[57]. There are several mechanisms to explain autoimmunity induced by infectious agents [58]. All are based on the observation that an immunocompetent host possesses circulating autoreactive T and B cells that are normally tolerant to self antigens [59]. As a consequence of the favorable proinflammatory environment induced by microorganisms, an unspecific activation may occur [60]. Excess BAFF may lower the threshold for BCR signaling and maintain survival when a normal autoreactive B cell would undergo death [61]. In agreement with reports showing evidences of BAFF participation in autoreactive response [62]–[67], BAFF blockade decreases the ANA IgG in *T. cruzi* infected mice. According to our results, it has been previously reported that a 4-week course of BAFF-R–Ig prevents the emergence of IgG anti-DNA antibodies in NZB/W mice [68].

The presence of anti-self antibodies was reported at the acute and chronic phase of *T. cruzi* infection [35], [37]. However, in our infection model, probably as consequence of different experimental conditions and the parasite and mouse strain used, we were unable to detect, by ELISA, anti-myosin, anti- myoglobin and anti-skeletal muscle antibodies in sera of acutely-infected mice (day 15 p.i.). Consequently, we ignore whether BAFF blockade affects the production of other autoreactive Abs different from ANA. Our data analyzed together indicated that autoreactive IgG3^+^ B cells activated during the infection and the parasite specific IgG^+^ B cells induced by *T. cruzi* show a differential requirement of BAFF to proliferate and/or differentiate and/or survive. This difference is probably related to the nature of B cell response: T-independent, extrafolicular or from germinal center [53]. Probably IgG3 ANA are produced during a pre-germinal center response [69]–[71] while anti-*T. cruzi* IgG Abs are produced in the course of a germinal center response (Bermejo et al. unpublished observation). Importantly, this differential requirement of BAFF may become an important target of manipulation to control a possible pathological autoreactive response without dampening the protective parasite specific response.

Interestingly, blocking BAFF is apparently not affecting the Abs involved in the control of circulating trypomastigotes. It seems that the conserved levels of IgG are sufficient to control parasite replication, or that, other populations different from B cells and not affected by BAFF inhibition, may be controlling parasite spreading. However, BAFF blockade does impact in *T. cruzi* replication in the heart, favoring the appearance of *T. cruzi* pseudocyts. The increase in the parasite replication in target tissues observed in BR3:Fc treated mice could be consequence of the markedly reduced mature B cell numbers that affect not only the production of Abs but also the development of protective cellular responses [72]. Strikingly, in spite of the high number of *T. cruzi* pseudocyts observed in the hearts of infected mice, preliminary data showed that BR3:Fc treated mice survive longer than non-treated infected mice (data not shown). Our findings enlighten a new role of BAFF-BAFF-R signalling in a parasite infection where it controls mature B cell numbers, polyclonal B cell activation and self-reactive response but does not affect protective anti-parasite IgG response.

## Supporting Information

Figure S1 Immature and mature B cell number in non-infected or *T. cruzi* infected mice treated with BR3:Fc. Cells from spleen and bone marrow from non-infected (NI) or *T. cruzi* infected (day 15 p.i.) mice treated with physiological solution (I) or BR3:Fc (I+BR3:Fc) or IgG2a control (I+IgG2a) were obtained. Cells from spleen were stained with anti-B220, anti-IgD and anti-IgM, and cells from bone marrow were stained with anti-B220, anti-IgD and anti-CD24 and analyzed by flow cytometry. Graphs show the number of: A) B220+ IgD+IgM+ (mature B cells) and B220+IgD-IgM+ in spleen and B) B220+IgD+CD24+ (mature B cells) and B220+IgD-CD24hi (immature B cells) in bone marrow. Diamonds represent the value obtained from each mouse. The lines represent the media value. *, p<0.05. Results are representative for three individual experiments.
(0.58 MB TIF)
