# Supplementary figures and images for: BAFF Mediates Splenic B Cell Response and Antibody Production in Experimental Chagas Disease
Source: PLoS Negl Trop Dis. 2010 May 4;4(5):e679. doi: 10.1371/journal.pntd.0000679 (PMC2864296; doi:10.1371/journal.pntd.0000679)

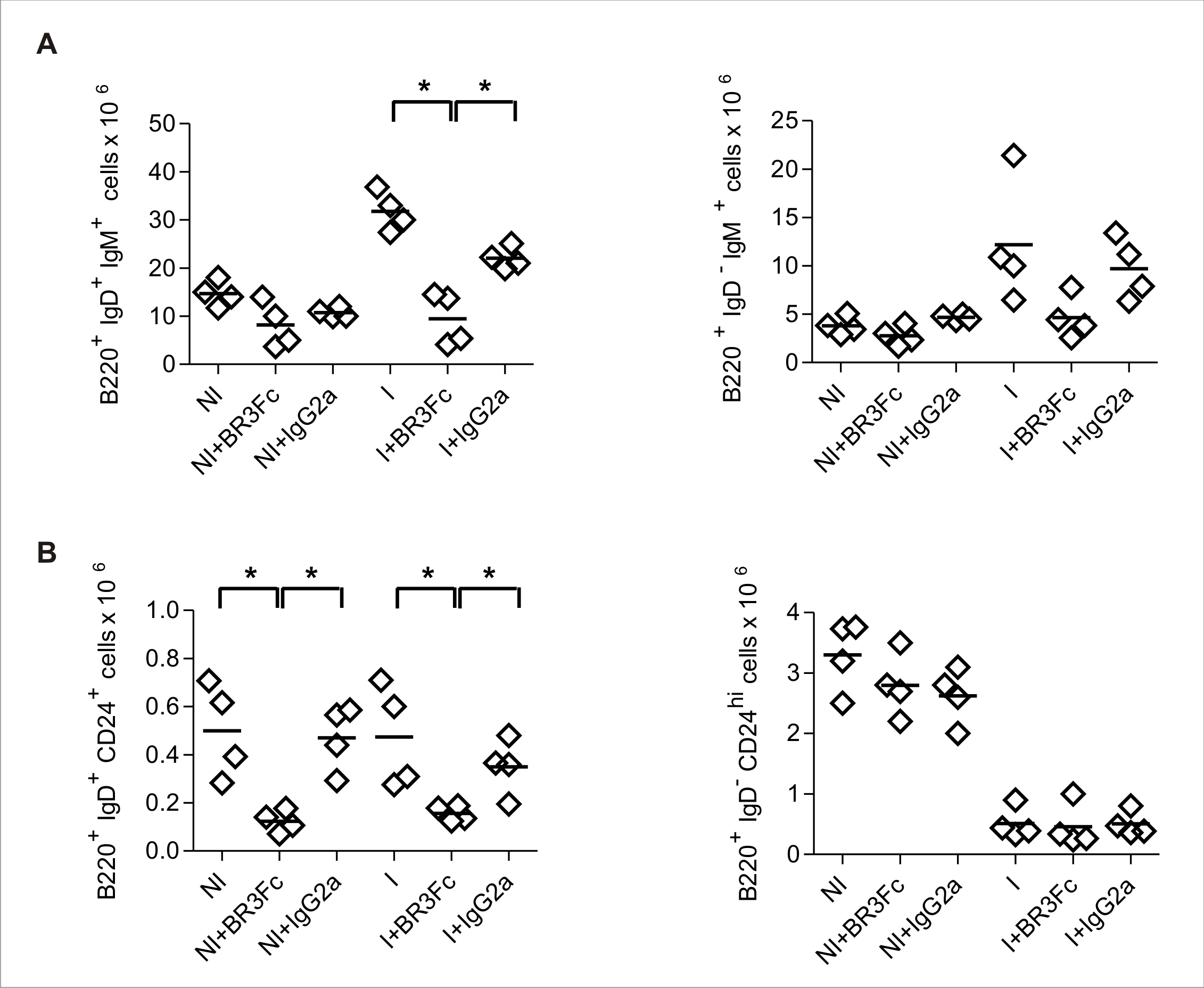

Supplement: Figure S1 — Immature and mature B cell number in non-infected or T. cruzi infected mice treated with BR3:Fc. Cells from spleen and bone marrow from non-infected (NI) or T. cruzi infected (day 15 p.i.) mice treated with physiological solution (I) or BR3:Fc (I+BR3:Fc) or IgG2a control (I+IgG2a) were obtained. Cells from spleen were stained with anti-B220, anti-IgD and anti-IgM, and cells from bone marrow were stained with anti-B220, anti-IgD and anti-CD24 and analyzed by flow cytometry. Graphs show the number of: A) B220+ IgD+IgM+ (mature B cells) and B220+IgD-IgM+ in spleen and B) B220+IgD+CD24+ (mature B cells) and B220+IgD-CD24hi (immature B cells) in bone marrow. Diamonds represent the value obtained from each mouse. The lines represent the media value. *, p<0.05. Results are representative for three individual experiments. (0.58 MB TIF) [file pntd.0000679.s001.tif]
